# Premature ventricular contractions with acute successful radiofrequency catheter ablation near the atrioventricular node using reversed C curve technique

**DOI:** 10.1186/s12872-022-02832-1

**Published:** 2022-08-31

**Authors:** Zhonghui Hu, Yunsheng Jiang, Su Wang

**Affiliations:** 1 Endocrine and Cardiovascular Department, Tianjin Fifth Central Hospital, No 41, Zhejiang Road, Tianjin, China; 2 Endocrine and Cardiovascular Department, Peking University Binhai Hospital, No 41, Zhejiang Road, Tianjin, China

**Keywords:** Catheter ablation, Reversed C curve technique, Ventricular arrhythmia, Tricuspid annulus, Atrioventricular node

## Abstract

**Background:**

We sought to clarify the electrophysiological (EP) characteristics of premature ventricular contractions (PVCs) with acute successful radiofrequency catheter ablation (RFCA) near the atrioventricular node (AVN).

**Methods and results:**

Eighteen patients with acute successful RFCA near the AVN were included in this study. Systematic mapping was performed with two mapping methods: antegrade mapping technique (group A) and reversed C curve mapping technique (group R). RFCA was preferentially performed underneath the tricuspid valve (TV) with reversed C curve technique in all patients. The a amplitude/v amplitude ratio during sinus rhythm in group A was significantly larger than in group B (0.19 ± 0.10 vs 0.06 ± 0.02, *p* < 0.01). The earliest bipolar activation preceded the QRS onset in group A was significantly smaller than in group R (19.6 ± 4.9 vs 24.4 ± 6.6 ms (ms), *p* < 0.01). Pace mapping in group A and group R demonstrated perfect QRS morphology (12/12) match only in 5.6% (one patient) and 16.7% (3 patients) of patients, respectively. The mean duration of successful RFCA was 8.2 ± 2.4 s in 13 patients (72.2%). Early (within 3 days) and late (one-year) recurrence rates were 5.6% (one patient) and 16.7% (3 patients), respectively. No atrioventricular block occurred during RFCA or the one-year follow up.

**Conclusions:**

PVCs near the AVN are a subgroup of idiopathic PVCs with distinctive EP features. RFCA using reversed C curve technique is effective and safe for the acute elimination of these challenging AVN-PVCs.

## Background

Most idiopathic ventricular arrhythmias (VAs), including idiopathic ventricular tachycardia (VT) and premature ventricular contractions (PVCs), have a right ventricular outflow tract (RVOT) or left ventricular outflow tract (LVOT) origin, but some may arise from other anatomical sites, including the right or left ventricular epicardial site, the aortic sinus cusps (ASCs), the aortomitral continuity (AMC), around the mitral annulus (MA), tricuspid annulus (TA), and other sites [1–3]. Radiofrequency catheter ablation (RFCA) or cryoablation has emerged as a treatment for these idiopathic PVCs near the atrioventricular node (AVN) or para-hisian region using antegrade RFCA technique [4–7]. RFCA of PVCs originating from near the AVN or para-hisian region (or more accurate to call them as presumed originating from underneath septal leaflet) can be frequently challenging because they have not only high risk of iatrogenic atrioventricular block (AVB), but also lower long-term success rates than other origins. What’s more, little is known about the prevalence, electrocardiographic (ECG) and electrophysiological (EP) characteristics, the efficacy of RFCA using reversed C curve technique and follow-up findings of AVN-PVCs. This study was undertaken to clarify these points.

## Methods

### Study participants

Among 454 consecutive patients who presented with premature ventricular contractions (PVCs) for RFCA between July 2000 and September 2018, 18 (3.96%) patients were found to have an acute successful radiofrequency catheter ablation (RFCA) of PVCs near the AVN. None of these patients exhibited significant coronary artery disease by coronary angiography or CT coronary angiography and any structural heart disease. They failed beta-blocker or at least one anti-arrhythmic drug therapy. All patients presented as PVCs manifesting left bundle branch block morphology with variable precordial transition (lead V_2_–V_5_). All patients were in normal sinus rhythm (SR) before RFCA. Twelve-lead ECGs and 24-h ambulatory Holter were carried out at least once before RFCA. The demographic and clinical data, including patient age, sex, height, weight, biochemical blood examination results, echocardiographic parameters and clinical arrhythmias, were collected prior to the procedure. The study protocol was reviewed and approved by the hospital’s ethics committee, and all patients provided written informed consent before undergoing RFCA.

### ECG analysis

All antiarrhythmic drugs were discontinued at least 5 half-lives before the ECGs were recorded for analysis. Twelve-lead ECGs leads were placed in the standard position and were recorded utilizing the Libang Electrical System (Libang ECG recording, Libang Medical, Shenzhen, China). The ECGs were analyzed at a paper speed of 25 mm/s, and the signals were amplified at 10 mm/mV. PVCs were analyzed for the following parameters: (1) The QRS duration; (2) The QRS amplitude in the limb leads; (3) The S-wave in lead V_5_/V_6_; (4) The amplitude of S wave in lead V_1_; (5) The precordial lead of R/S > 1; (6) The QRS complex duration during PVC and SR. All parameters were measured with electronic calipers by 3 experienced investigators blinded to the site of origin. The mean values of these measurements were used for analysis. If the inter-observer difference was more than 5 ms, a final decision was adjudicated by a joint meeting of the three investigators.

### Preparation and activation mapping

All antiarrhythmic drugs were discontinued at least 5 half-lives before the EP study. Intracardiac tracings were recorded utilizing a Prucka CardioLab™ Electrophysiology System (General Electric Health Care System, Inc, Milwaukee, WI, USA). If the clinical PVCs did not occur spontaneously and were not induced at baseline, intravenous isoproterenol (0.5 to 2.0 g/min) was administered to induce the clinical PVCs. A 7.5-French, 3.5-mm-tip, irrigated ablation catheter (F-curve, NaviStar ThermoCool, Biosense Webster, Diamond Bar, CA, USA) was then introduced into the right ventricle (RV) using a steerable sheath (MobiCath Large curve, Biosense Webster, Diamond Bar, CA, USA) via the right femoral vein, and intravenous heparin was administered to maintain an activated clotting time of 200–250 s (s). Defining earliest activation near the AVN requires detailed mapping of the His bundle, coronary sinus ostium, as well as the adjacent inferomedial TV together with the adjoining atrial myocardium. Mapping was all initiated with antegrade technique and then with reversed C curve technique underneath the septal leaflet of the TV for the 18 cases. The surrounding sites were also mapped: tricuspid annulus, proximal coronary sinus or coronary sinus diverticulum, intraventricular septum, outflow tracts, aortic cusps, posterior-superior process, and mitral annulus.

### Electrogram (EGM) collection and analysis

During an episode of spontaneous clinical PVCs, activation mapping was performed on at least three arrhythmic beats at a mapping site. The unipolar EGM was recorded from the distal (D) electrode of the mapping catheter and filtered at 0.5–100 Hz. The bipolar EGM was recorded from the distal (D-2) electrode pairs of the mapping catheter and filtered at 30–500 Hz. All EGM and twelve-lead ECG data were stored on the multichannel mapping system for offline analysis with a paper speed of 100 mm/s [8]. The V-QRS interval was calculated from the start of the bipolar ventricular EGM to the earliest start of QRS complex from any lead (QRS from surface leads and EGM from all intra-cardiac channels). All parameters were measured with electronic calipers by 3 experienced investigators blinded to the site of origin. We adopted the mean values of these measurements as the data. If the inter-observer difference was more than 5 ms, a final decision was adjudicated by a joint meeting of the three investigators.

### Pace mapping

Pace mapping during SR was performed at the earliest activation site using the distal bipolar electrodes at a coupling interval of the PVCs interval and a stimulus amplitude of 1 mA greater than the late diastolic threshold (up to a maximum output of 10 mA and pulse width of 2.0 ms). Pacing was done both with antegrade technique and reversed C curve technique. If present, a perfect pace-mapping match (11/12 or 12/12 leads) was indicative of the site at or in close proximity to the PVCs origin; otherwise, the activation mapping result was only used for guiding RFCA.

### RFCA

RFCA was applied at the site where the earliest V-QRS interval or perfect pace-mapping match on ECG was recorded with reversed C curve technique. RFCA was delivered using the power-control mode to this site starting from 20 W and was cautiously increased to 40 W with a temperature of 43 °C using irrigation mode at a flow rate of 17 mL/min. If PVC suppression or acceleration was observed during the first 15 s (s) of RFCA application, the energy application was continued for a total of 90–120 s at and around the target site while monitoring for junctional rhythm and antegrade atrioventricular conduction, and the site was tagged as a successful site on the map. Otherwise, RFCA delivery was terminated and the catheter was repositioned.

### Definition of acute successful RFCA

Acute successful RFCA was defined according to the following criteria: absence of spontaneous or induced clinical PVCs, including a stimulation protocol (ventricular and/or atrial pacing) and intravenous isoproterenol infusion after RFCA with observation time lasting from 30 to 60 min.

### Definition of near AVN origin

We defined a near AVN origin as follows: (1) The catheter tip demonstrated the tricuspid annulus (TA) septal side when viewed on the right and left anterior oblique fluoroscopic views at the successful RFCA site; (2) A far field atrial EGMs could be recorded at the RFCA site during SR with reversed C curve mapping technique with a amplitude/v amplitude ratio less than 0.1; (3) Acute successful RFCA location was achieved at the midway between the His potential and coronary sinus ostium; (4) Acute successful PVCs elimination was achieved by RFCA energy delivery at the RFCA location.

### Observation after RFCA and at the one-year follow up

The patients were monitored for at least 3 days in the hospital after RFCA, and twelve-lead ECG and 24-h ambulatory Holter monitoring were carried out at least once during the 3-day hospitalization after RFCA. The patients were followed up in the outpatient arrhythmia clinic for one year, and twelve-lead ECG and 24-h ambulatory Holter monitoring were carried out at least once every three months. Clinical success was defined as absence of PVCs of the same morphologies that were targeted during the ablation procedure confirmed by 12-lead ECG in patients who came for a follow-up visit and/or 80% reduction of PVC burden on post-RFCA monitor compared to pre-RFCA PVC burden.

### Statistical analysis

Continuous data are given as the mean ± SD, differences in continuous variables were assessed using paired-samples T test or one-way ANOVA, categorical variables were assessed using Fisher’s Exact Test. A p value < 0.05 was considered to indicate statistical significance. Statistical analyses were performed using the SPSS software (Version 11.0, SPSS, Chicago, IL, USA).

## Results

### Location and frequency of PVCs

Of 454 patients referred for RFCA of idiopathic PVCs, eighteen (3.96%) patients had successful RFCA near the AVN region. The other origins were registered as follows: RVOT, 42.73%; ASC, 29.30%; AMC, 7.49%; mitral annulus, 11.01%; tricuspid annulus except for near the AVN region, 2.20%; papillary muscle, 3.08%; and possible right ventricular moderate band, 0.22%. The mean age of the 18 patients was 68.3 ± 9.7 years with 13 males and 5 females, and their clinical characteristics were summarized in Table 1.

**Table 1:** Characteristics of the study population

	N = 18
Age (years)	68 ± 10 (51–79)
Male sex (%)	13/18 (72.2%)
Height (cm)	166.4 ± 8.1
Weight (kg)	71.8 ± 13.9
K (mmol/L)	4.2 ± 0.3
Cr (μmol/L)	67.4 ± 12.0
UA (μmol/L)	313.3 ± 89.2
RA (mm)	34.8 ± 6.3
RV (mm)	32.4 ± 4.4
LA (mm)	37.4 ± 3.9
LV (mm)	47.3 ± 4.6
LVEF (%)	62.6 ± 5.0
History (years)	3.4 ± 4.9*
PVC load (%)	17.5 ± 5.9
Clinical VAs	
Only PVC	18/18 (100%)
PVC, nonsustained VT	0/18 (0%)
VAs episode	
Frequently episode of clinical VAs during baseline (%)	14/18 (77.8%)
Needs isoproterenol infusion to induce clinical VAs (%)	4/18 (22.2%)

Values are given as the mean ± SD or n (%), unless otherwise indicated. * Indicates non-normally distributed data

### ECG characteristics of AVN-PVCs

**Fig. 1:**
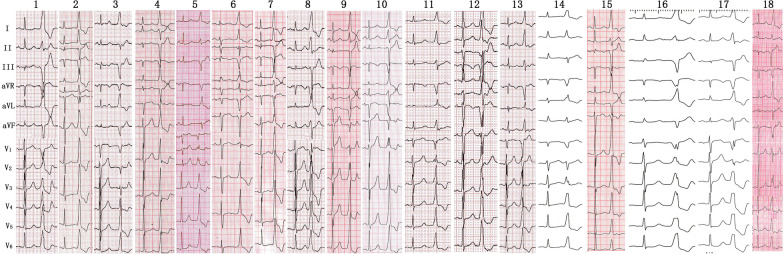
Twelve-lead electrocardiographic (ECG) QRS morphology of all 18 patients The first complex is sinus rhythm (SR) and the second is premature ventricular contraction (PVC). (25 mm/s speed)

During the clinical PVCs, the R_I_, R_II_, S_II_, R_III_, S_III_, R_aVR_, R_aVL_, R_aVF_, R_aVF_, S_aVF_, and S_V1_ amplitudes were 1.24 ± 0.34 mV, 0.66 ± 0.32 mV, 0.17 ± 0.18 mV, 0.13 ± 0.33 mV, 1.06 ± 0.54 mV, 0.79 ± 0.24 mV, 1.07 ± 0.39 mV, 0.25 ± 0.22 mV, 0.62 ± 0.32 mV, 0.74 ± 0.28 mV, respectively (Table 2). In all cases, leads I and aVL had positive forces and lead aVR had negative forces (Fig. 1). The QRS duration during PVCs and SRs were 123.6 ± 7.57 ms and 90.9 ± 13.5 ms, respectively. Precordial R/S > 1 transition in Lead V_2_, V_3_ and V_4_ and S-waves in lead V_5_/V_6_ were recorded in 50%, 33.3%, 16.7% and 0% of patients, respectively. QRS morphology in lead V_1_ of Qrs, QS, Qr pattern were recorded in 50%, 33.3%, %, 16.7%, of patients, respectively.

**Table 2:** ECG Characteristics of PVCs with acute successful RFCA near the AVN

	N = 18
QRS duration during VAs (ms)	123.6 ± 7.57
QRS duration during sinus rhythm (ms)	90.9 ± 13.5
r amplitude in lead I (mV)	1.24 ± 0.34
r amplitude in lead II (mV)	0.66 ± 0.32
s amplitude in lead II (mV)	0.17 ± 0.18*
r amplitude in lead III (mV)	0.13 ± 0.33*
s amplitude in lead III (mV)	1.06 ± 0.54
q amplitude in lead aVR (mV)	0.79 ± 0.24
r amplitude in lead aVL (mV)	1.07 ± 0.39
r amplitude in lead aVF (mV)	0.25 ± 0.22
s amplitude in lead aVF (mV)	0.62 ± 0.32
s amplitude in lead V_1_ (mV)	0.74 ± 0.28
QRS morphology in lead V_1_	
Qrs (%)	9/18 (50%)
QS (%)	6/18 (33.3%)
Qr (%)	3/18 (16.7%)
Precordial R/S > 1 in Lead V_2_	9/18 (50%)
Precordial R/S > 1 in Lead V_3_	6/18 (33.3%)
Precordial R/S > 1 in Lead V_4_	3/18 (16.7%)
Slurred onset of the precordial QRS complexes	13/18 (72.2%)
S wave in lead V_5_/V_6_ (%)	0/18 (0%)

Values are given as the mean ± SD or percent (%), unless otherwise indicated. * Indicates non-normally distributed data

### Activation mapping of clinical PVCs

**Fig. 2:**
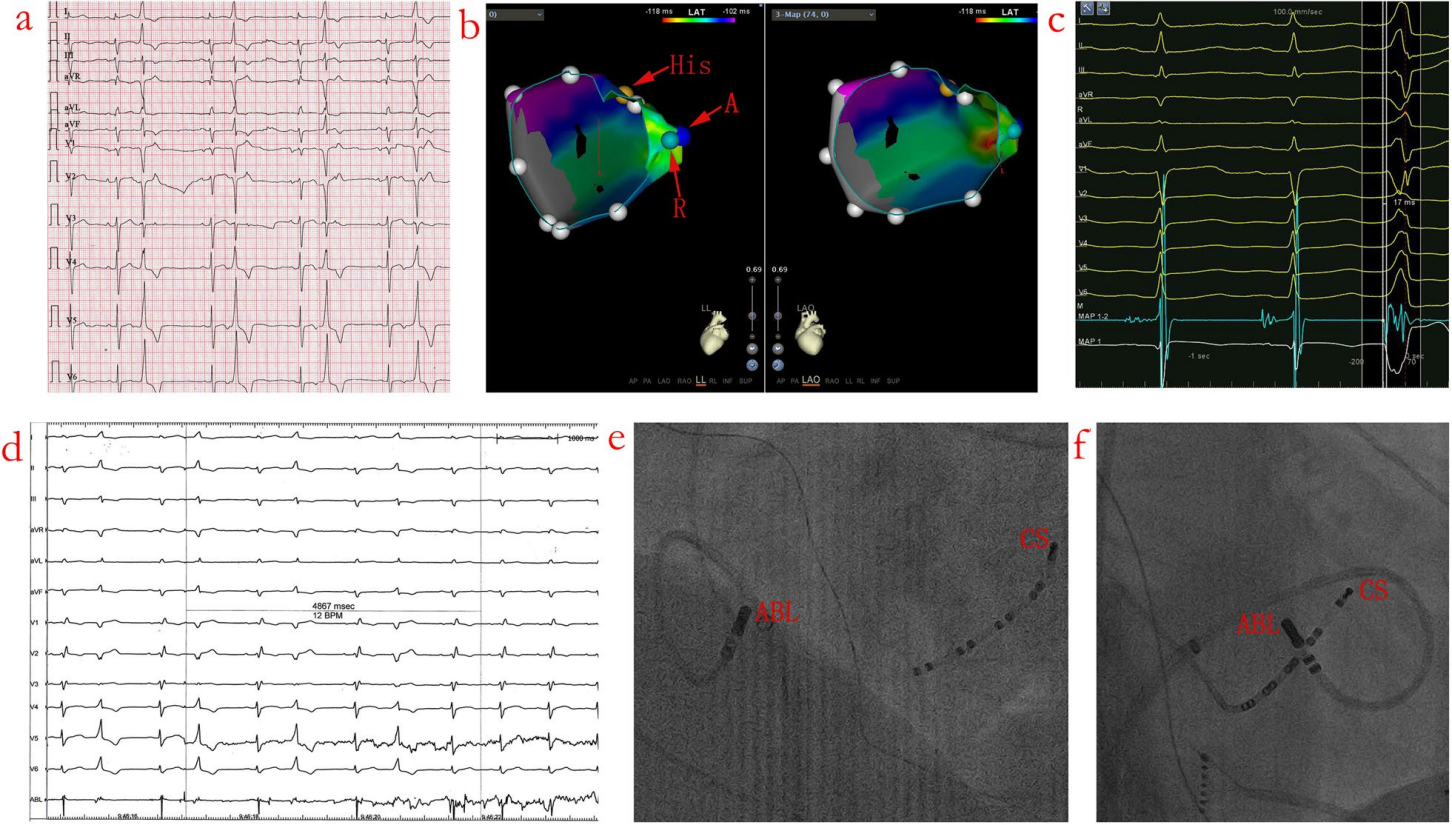
PVC with acute successful ablation near the atrioventricular node (AVN) (Patient No 6). **a** Twelve-lead ECG morphology of the QRS complex during SR and PVC. **b** CARTO3 mapping indicates an acute successful RFCA site near the AVN (A stands for antegrade technique and R stands for reversed C curve technique, yellow dot is where local electrogram has prominent his potentials in sinus beats). **c** Earliest V-QRS interval of 17 ms for bipolar recording during PVC, initial Q wave for unipolar recording, near field atrial electrogram (EGM) and an a/v amplitude ratio of 0.10 during SR (paper speed 100 mm/s), consistent with the recording from near the AVN with antegrade technique. **d** RFCA leads to elimination of the PVC after 4.87 s ablation delivery with reversed C curve technique. **e** and **f** Left and right anterior oblique fluoroscopic views indicate reversed C curve mapping technique. See the text for further details (paper speed 25 mm/s unless indicated). CS = coronary sinus; ABL = ablation catheter; MAP 1–2 = bipolar recording; MAP 1 = unipolar recording; Stim = stimulation. The same explanation as in Fig. 3 unless indicated

**Fig. 3:**
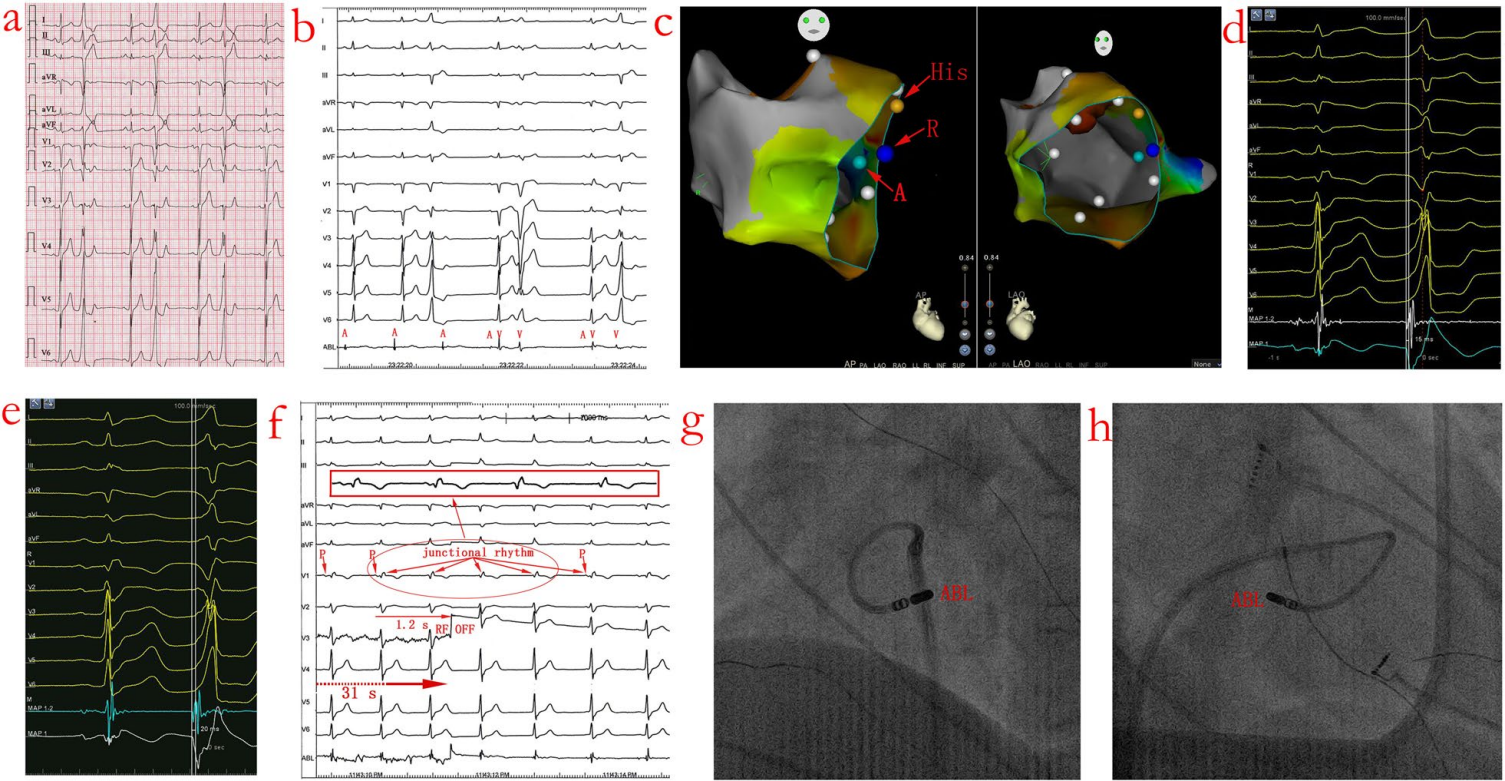
PVC with acute successful ablation neat the AVN (Patient No 10). **a** Twelve-lead ECG morphology of the QRS complex during SR and PVC. **b** Catheter manipulation during reversed C curve mapping lead to functional right bundle branch block. **c** CARTO3 mapping indicates an acute successful RFCA site near the AVN. **d** Earliest V-QRS interval of 15 ms for bipolar recording during PVC, initial Q wave for unipolar recording, near field atrial EGM and an a/v amplitude ratio of 0.08 during SR (paper speed 100 mm/s), consistent with the recording from near the AVN with antegrade technique. **e** Earliest V-QRS interval of 20 ms for bipolar recording during PVC, initial Q wave for unipolar recording, far field atrial EGM and an a/v amplitude ratio of 0.08 during SR with reversed C curve technique (paper speed 100 mm/s). **f** Junctional rhythm was noted during RFCA energy application of 31 s in this location and RFCA was discontinued 1.2 s later. **g** and **h** Left and right anterior oblique fluoroscopic views indicate reversed C curve technique. See the text for further details (paper speed 25 mm/s unless indicated)

Detailed mapping of the middle cardiac vein, coronary sinus, right and left sides of the basal ventricular septal regions were performed in all the 18 patients. In all cases, no areas of abnormal endocardial voltage were seen in both right and left sides of the basal ventricular septal regions. Acute successful RFCA near the AVN was achieved in all 18 patients with a reversed C curve technique. As shown in Table 3, the a amplitude/v amplitude ratio during SR in group A was significantly larger than in group B (0.19 ± 0.10 vs 0.06 ± 0.02, *p* < 0.01). The earliest bipolar activation preceded the QRS onset in group A was significantly smaller than in group R (19.6 ± 4.9 vs 24.4 ± 6.6 ms, *p* < 0.01). Initial unipolar QS-waves were recorded in 16 patients (88.9%) in group A and in 17 patients (94.4%) in group R. Visible His potential were recorded in 5 patients (27.8%) in group A and 2 patients (11.1%) in group B during SR. With bipolar mapping, isolated pre-potential, low voltage fragmented potential, earliest V-QRS interval without isolated pre-potential or low voltage fragmented potential preceding the QRS complexes was recorded in 0 (0%), 4 (22.2%), 14 (77.8%) of patients in group A and 0 (0%), 5 (27.8%), 13 (72.2%) of patients in group B, respectively. Activation mapping with antegrade technique/reversed C curve technique in Figs. 2c and 3c showed V-QRS interval of 17 ms/25 ms and 15 ms/20 ms for bipolar recording during PVCs.

**Table 3:** Comparison of the two mapping methods

	Antegrade technique (group A, n = 18)	Reversed C curve technique (group R, n = 18)	t/χ^2^ values	*p* values
a amplitude/v amplitude ratio during sinus rhythm	0.19 ± 0.10	0.06 ± 0.02	5.17	0.000
Earliest bipolar V-QRS interval during clinical VAs (ms)	19.6 ± 4.9	24.4 ± 6.6	3.20	0.005
Initial QS wave during uniplolar recording	16/18 (88.9%)	17/18 (94.4%)	–	1
Visible His potential recorded	5/18 (27.8%)	2/18 (11.1%)	–	0.402
Target characteristics				
Isolated pre-potential (%)	0/18 (0%)	0/18 (0%)	–	1
Low voltage fragmented potential (%)	4/18 (22.2%)	5/18 (27.8%)	–	1
Non low voltage fragmented potential (%)	14/18 (77.8%)	13/18 (72.2%)	–	1
Pace mapping				
Captured a (%)	6/18 (33.3%)	0 (0%)	–	0.190
Intermittent captured a or v (%)	3/18 (16.7%)	0 (0%)	–	0.229
Captured v (%)	7/18 (38.9%)	18 (100%)	–	0.000
No capture (%)	2/18 (11.1%)	0 (0%)	–	0.000
Perfect pace mapping with 11/12 or 12/12	1/18 (5.6%)	3/18 (16.7%)	–	0.603
Lead to functional right branch bundle block	0 (0%)	12/18 (66.7%)	–	0.000

Values are given as the mean ± SD (range) or n (%), unless otherwise indicated

### Pace mapping

Perfect (12/leads) or near perfect (11/12 leads) pace maps to the QRS morphology of the clinical PVCs were obtained in 1 patient (5.6%) and 3 patients (16.7%) in group A and group B, respectively. Table 3 showed antegrade technique for pace mapping of captured atrium, intermittent captured atrium or ventricle, captured ventricle, no capture of atrium and ventricle in 33.3%, 16.7%, 38.9%, 11.1% of patients, respectively. A relatively large amount of myocardium could be captured around the pacing electrodes, which might have obscured subtle or obvious changes in the QRS morphology in most of these patients.

### RFCA underneath the septal leaflet of the TV

Complete elimination of the PVCs could be achieved by RFCA at the site where the earliest V-QRS interval was recorded with reversed C curve technique underneath the septal leaflet of the TV. The mean duration of successful RFCA was 8.2 ± 2.4 s in 13 patients (72.2%). In the remaining 5 patients (27.8%), the mean duration of successful RFCA was not well determined due to infrequent nature of clinical PVCs during ablation. Junctional rhythm was recorded in 2 patients (11.1%) after 28 and 31 s of RFCA, respectively. RFCA was carried out only intermittently and was discontinued immediately when junctional rhythm occurred. The atrioventricular (AV) 1:1 antegrade conduction intervals before and after RFCA were 389.0 ± 36.4 ms and 401.3 ± 37.1 ms (*p* = 0.009), but without manifest PR prolongation during SR. No complications including AVB, pericardial effusion occurred during the RFCA procedure. Figure 3f showed transient junctional rhythm during RFCA to 31 s and RFCA was discontinued 1.2 s later. The successful reversed C curve technique for RFCA target location was shown in Figs. 2e/f and 3g/h (left and right anterior oblique fluoroscopic views).

### Observations after RFCA and at the one-year follow up

No complications occurred during at least 3 days of observation after the index procedure or during the one-year follow-up period. Clinical PVCs could still be recorded in 1 patient (5.3%) during 3 days of in-hospital monitoring after RFCA. During the one-year follow-up period, 3/18 (16.7%) patients had clinical PVCs recurrence based on the same QRS morphologies during VAs, and one patient undergo re-do procedure. The final RFCA target was the same as the index procedure based on X-ray fluoroscopic views and CARTO3 mapping result.

## Discussion

### Main findings

The current study has four major findings. First, the frequency of PVCs near the AVN confirmed by acute successful RFCA was near 4% in 454 consecutive patients with idiopathic PVCs in a single center. Second, PVCs near the AVN can be identified by their unique ECG features, precordial R/S > 1 by V_2_ or V_3_ (over 80%) and discordant QRS patterns in inferior leads with R_II_ > R_III_ and S_III_ > S_II_, which is the distinctive ECG features in this single center study. Third, all PVCs near the AVN were with acute successful RFCA underneath the septal leaflet of the tricuspid valve (TV). Forth, the reversed C curve technique, as compared to the antegrade technique, showed a smaller local a/v ratio during sinus rhythm (SR) and earlier local bipolar V-QRS interval with great safety and success rate in eliminating PVCs near the AVN.

### ECG characteristic of PVCs from near the AVN

The origin of PVCs near the AVN is located underneath the septal leaflet of the TV. PVCs originating from this region can manifest variable ECG morphologies. The myocardium at the RFCA site is depolarized in a direction away from lead III and toward lead II. This could account for the significantly larger S_III_ than S_II_ and S_aVF_, and larger R_II_ than R_III_ and R_aVF_. The myocardium at the RFCA site depolarize from right ventricle to left ventricle which may explain the negative morphologies in lead V_1_, V_2_, V_3_ and (or) V_4_, different precordial R/S > 1 transition in different patients and the absence of an s-wave in lead V_5_/V_6_, depending on the exit site (Table 2 and Fig. 1). The characteristic ECG findings for AVN-PVCs in the present study are useful for electrophysiologists in formulating a strategy and preparing instruments for RFCA to maximize the ease and likelihood of success of the procedure.

### Antegrade technique versus reversed C curve technique and RFCA

Both activation mapping and pace mapping methods were performed and compared for the potential characteristics with antegrade technique and reversed C curve technique. Anatomical and technical difficulties such as poor catheter contact and instability due to mobile septal leaflets of the TV with antegrade technique and higher risk of AVB, which precludes this antegrade ablation method. RFCA application with antegrade RFCA technique resulting in PVC suppression with junctional rhythm indicate that the AVN was at least the exit site if not the site of PVC origin [4]. Instead, we demonstrated in this study that a feasible solution using a reversed C curve technique could improve the success rate and decrease the possible hazards especially AVB during RFCA of AVN-PVCs. Catheter manipulation during reversed C curve technique mapping and (or) RFCA lead to functional right bundle branch block in 12 of 18 patients (66.7%) but all recovered during 3 days of in-hospital monitoring (Fig. 3e and Table 4). We could not exclude the possibility that some PVCs may be intramural in the inferior septal location near the AVN. This was suggested, although not proven, by the need for higher RF energy and longer duration of RFCA for successful elimination of PVCs in some cases. Alternatively, the pacing techniques reported by Luo S et al. may help to differentiate the far field and near field His potential and decrease the risk of AVB [9].

**Table 4:** Ablation and follow up result of PVCs originating from near the AVN

	N = 18
Start to effect time (s)	8.2 ± 2.4#
Total ablation time (s)	487 ± 127
Procedure time (h)	2.3 ± 1.1
Junction rhythm during ablation (%)	2/18 (11.1%)
AV 1:1 antegrade conduction before ablation (ms)	389.0 ± 36.4
AV 1:1 antegrade conduction after ablation (ms)	401.3 ± 37.1*
Recurrence during 3 days of in-hospital monitoring (%)	1/18 (5.6%)
Permanent CRBBB existed during 3 days of in-hospital monitoring (%)	0/18 (0%)
Recurrence during one-year follow up (%)	3/18 (16.7%)

Values are given as the mean ± SD (range) or n (%), unless otherwise indicated. *: compared with AV 1:1 conduction before ablation group, t = 2.97, *p* = 0.009. CRBBB: right branch bundle block recurred. #: The mean duration of successful RFCA time were for the 13 patients (72.2%), in the remaining 5 patients (27.8%), the mean duration of successful RFCA was not well determined due to infrequent nature of clinical PVCs during ablation

### Anatomical consideration

**Fig. 4:**
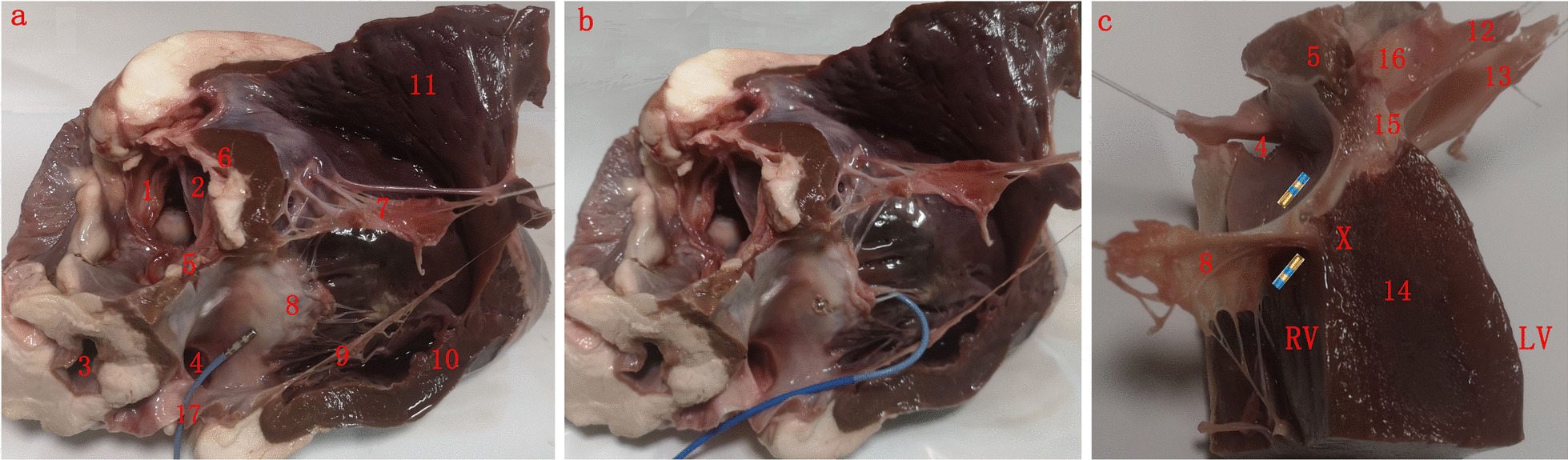
Heart specimens from a bovine heart. **a** Antegrade technique demonstrating the relationships between the septal leaflet of tricuspid valve (TV) and ablation catheter orientation. **b** Reversed C curve technique demonstrating the relationships between the septal leaflet of TV and ablation catheter orientation. **c** Nonattitudinal frontal transection through the interventricular septum at the level of ablation location illustrating the anatomical relations and direction of the ablation catheter according to technique route. “X” denotes the hypothetical arrhythmia focus at the muscular crest. 1, left coronary cusp; 2, right coronary cusp; 3, right pulmonary vein; 4 coronary sinus; 5, atrial septum; 6, right coronary artery; 7, anterior leaflet of TV; 8, septal leaflet of TV; 9, posterior leaflet of TV; 10, right ventricular free wall; 11, right ventricular output tract free wall; 12, non-coronary leaflet; 13, posterior leaflet of mitral valve; 14, ventricular septum; 15, central fibrous body; 16, non-coronary cusp; 17, inferior vena cava; RV, right ventricle; LV, left ventricle

The septal leaflet of the TV has very important anatomical relationship with the His bundle, which is located within the membranous ventricular septum [10–12]. After the membranous septum, His bundle continues downward as right and left bundle branches over the muscular crest of ventricular septum. The septal leaflet of the TV completely covers the crest of inter-ventricular muscular septum from where AVN-PVCs are presumably originating. This specific anatomical region has very important practical implications with antegrade RFCA application is that the septal leaflet of the TV may act as a heat shield and prevent effective RFCA application on the muscular septum (Fig. 4). This may explain, even though the activation map “seemingly” perfect, the PVCs typically disappear transiently upon RFCA application and reappear after the energy delivery is stopped. Another feasible option would be selecting a retrograde C curve with an inverted catheter aiming to reach the subvalvular area in order to contact directly with basal muscular septum. RFCA application would be more likely successful in eliminating the PVCs with relatively low risk of injury to the leaflet and AVN antegrade conduction (Table 4).

### Related studies

Several previous studies have reported the successful ablation of PVCs near the AVN region. However, none has systemically determined the prevalence, ECG and EP characteristics of PVCs originating from near the AVN region and short and long-term RFCA outcomes. Van Herendael et al. reported the same 12-lead ECG morphology near the AVN when pacing the septal portion of the valvular aspect of the right ventricle (RV) [13]. The inferior lead discordance observed in our series has also been previously noted by Enriquez et al. and Briceño DF et al. [4, 14]. Briceño EF et al. reported RFCA by antegrade technique resulting in junctional rhythm in 9 (75%) and successful ablation in 11 patients (92%). One patients developed only transient AVB, and another developed high-grade AVB requiring a pacemaker [4]. Sun J et al. reported catheter ablation of ventricular arrhythmias originating from the para-Hisian region with reversed C curve technique, during a mean follow‐up of 17.8 ± 9.4 months, no patients presented with VAs recurrence and no post procedure complications especially AVB occurred [15]. Miyamoto et al. reported that cryoablation can achieve acute success rate of 70% (7/10) for para-Hisian PVCs by antegrade technique. However, cryoablation also lead to AVB for 1 patient during the cryoenergy application and a permanent pacemaker was implanted [7]. The septal tricuspid valve completely covers the membranous septum and causes an anatomic obstacle for ablation of para‐Hisian VAs, thus, it is not surprising that supravalvular catheter ablation with the traditional antegrade method has a relatively low success rate and high recurrence rate. Bipolar RFCA is an alternative method to treat para‐Hisian Vas, however, performing biplolar-RFCA requires perpendicular orientation of both catheters, which can be challenging and may require assistance of a second electrophysiologist [16]. All of these studies suggest that ablation of these PVCs is challenging, for either RFCA or cryoablation.

### Possible mechanisms of PVCs near the AVN

While the mechanism of AVN-PVCs cannot be determined from a clinical study, an appreciation of the particular anatomy and the lack of overlying connective muscular tissue of the distal compact AVN and proximal His bundle potentially provides some insights [15, 17, 18]. Different EP characteristics between the AVN-His conduction system and the neighbouring ventricular myocardium which practically may potentially provide the anisotropic conduction necessary for the initiation of re-entry, abnormal automaticity or triggered activity, was speculated as to the role of these tissues in ventricular arrhythmogenesis.

### Clinical implications

Catheter ablation of AVN-PVC is a challenging task and has a risk of AVB. The distal compact AVN and proximal His bundle have no overlying connective muscular tissue layer which practically makes them relatively unprotected to RFCA injury. While in the subvalvular region, the distal His bundle is covered and well protected by an insulating layer. RFCA using the reversed C curve technique in the subvalvular region shows to be safe for RFCA of these AVN-PVCs with lower risk of damage to conduction system because the AVN is protected by the TV during RFCA application and distal His bundle is well protected by insulating layer at this location [15, 17, 18].

## Study limitations

There are several limitations with this retrospective study. First, intra-cardiac echocardiography was not used to confirm the RFCA target. Therefore, whether the exact PVCs RFCA target are underneath the septal leaflet of the TV or not could not be ascertained. Second, the genesis of PVCs near the AVN were not determined. Third, all of the PVCs were successfully ablated despite that isolated pre-potential or low voltage fragmented potentials during PVCs were not recorded. The causes for lack of recording for isolated pre-potential or fragmented potential were only speculative. Fourth, cryoablation with reversed C curve technique could be difficult to achieved the subvalvular area due to the stiffness of the cryoablation catheter. Fifth, this was a retrospective analysis result, which needs to be validated with a prospective study with a larger sample size.

## Conclusions

PVCs near the AVN are a subgroup of idiopathic PVCs with distinctive ECG and EP features. RFCA with reversed C curve technique underneath the septal leaflet of the TV is effective and safe for the acute elimination of these challenging AVN-PVCs. Advanced knowledge of the anatomy near the AVN, His bundle and TV, ECG and EP features are useful in planning and facilitating the RFCA procedure.

## Data Availability

The datasets used and/or analyzed during the current study are identified and available from the corresponding author on reasonable request. All data generated during this study are included in this published article.

## Abbreviations

VAs: Ventricular arrhythmias
VT: Ventricular tachycardia
PVC: Premature ventricular contraction
RFCA: Radiofrequency catheter ablation
AVN: Atrioventricular node
RVOT: Right ventricular outflow tract
LVOT: Left ventricular outflow tract
ASC: Aortic sinus cusps
AMC: Aortomitral continuity
MC: Mitral annulus
TA: Tricuspid annulus
AVB: Atrioventricular block
ECG: Electrocardiographic
EP: Electrophysiology
SR: Sinus rhythm
TV: Tricuspid valve
RV: Right ventricle
